# The Unequal Taxonomic Signal of Mosquito Wing Cells

**DOI:** 10.3390/insects12050376

**Published:** 2021-04-21

**Authors:** Somsanith Chonephetsarath, Chadchalerm Raksakoon, Suchada Sumruayphol, Jean-Pierre Dujardin, Rutcharin Potiwat

**Affiliations:** 1 Department of Medical Entomology, Faculty of Tropical Medicine, Mahidol University, Bangkok 10400, Thailand; somsanithchonephetsarath@gmail.com (S.C.); suchada.sum@mahidol.ac.th (S.S.); 2 Department of Chemistry, Faculty of Science, Kasetsart University, Bangkok 10900, Thailand; fsciclr@ku.ac.th; 3 Institut de Recherche pour le Développement (IRD), UMR INTERTRYP IRD-CIRAD, University of Montpellier, F-34398 Montpellier, France; jean-pierre@xyom.io

**Keywords:** geometric morphometrics, outlines, *Aedes aegypti*, *Aedes albopictus*, *Aedes scutellaris*, *Verrallina dux*

## Abstract

**Simple Summary:**

Mosquitoes of the genus *Aedes* include important vectors of human disease viruses, including dengue, chikungunya and Zika. Surveillance programs used to detect and control these pests need accurate, fast and low-cost techniques to track the primary target and monitor possible re-infestations. Geometric morphometrics of mosquito wings is a convenient tool in mosquito species identification, but this method requires a complete wing in good condition for maximum accuracy. In this study, we investigate the amount of taxonomic signal provided by shape analysis of the internal cells of the wing. We show that (i) the internal cells of the wing provide differing amounts of taxonomic information, and (ii) the taxonomic signal of a given cell depends on the species under comparison. Since some of these cells are very informative, our study suggests that even damaged wings may provide key taxonomic information to differentiate among species found in mixed species surveillance collections.

**Abstract:**

Accurate identification of mosquito species is critically important for monitoring and controlling the impact of human diseases they transmit. Here, we investigate four mosquito species: *Aedes aegypti*, *Ae. albopictus*, *Ae. scutellaris* and *Verrallina dux* that co-occur in tropical and subtropical regions, and whose morphological similarity challenges their accurate identification, a crucial requirement in entomological surveillance programs. Previous publications reveal a clear taxonomic signal embedded in wing cell landmark configuration, as well as in the external contour of the wings. We explored this signal for internal cells of the wings as well, to determine whether internal cells could uniformly provide the same taxonomic information. For each cell to be tentatively assigned to its respective species, i.e., to measure the amount of its taxonomic information, we used the shape of its contour, rather than its size. We show that (i) the taxonomic signal of wing shape is not uniformly spread among internal cells of the wing, and (ii) the amount of taxonomic information of a given cell depends on the species under comparison. This unequal taxonomic signal of internal cells is not related to size, nor to apparent shape complexity. The strong taxonomic signal of some cells ensures that even partly damaged wings can be used to improve species recognition.

## 1. Introduction

Many mosquito species are known vectors of virus, including *Aedes (Stegomyia) aegypti* (Linnaeus *in* Hasselquist, 1762), *Ae. (St.) albopictus* (Skuse, 1894) and *Ae. (St.) scutellaris* (Walker, 1859). The traditional supraspecific arrangement of these three species, initially belonging to the *Aedes* genus, was tentatively modified two decades ago, restoring the genus *Stegomyia* [1], and suggesting the names *Stegomyia aegypti*, *St. albopicta* and *St. Scutellaris* [1,2]. In this study, for these three well-known vectors and following their common use among epidemiologists [3], we will use the traditional genus and subgenus name *Aedes (Stegomyia)*. A fourth species discussed here belongs to the genus *Verrallina* (redefined by Reinert, 1999) [4], subgenus *Verrallina*: *Ve. (Ver.) dux* (Dyar and Shannon, 1925) [2].

*Aedes aegypti* (Linnaeus in Hasselquist, 1762), *Ae. Albopictus* (Skuse, 1894) and *Ae. scutellaris* (Walker, 1859) are vectors of various viruses [5,6]. The two first species, *Ae. aegypti* and *Ae. albopictus,* have a wide intercontinental distribution, including in Thailand, where they recently contributed to an important dengue and chikungunya viral outbreak [7,8]. In addition to transmitting the chikungunya virus, they are also able to spread all of the four dengue serotypes (DENV1-4) [9]. *Aedes aegypti* is also a competent vector of Zika virus [10]. *Ae. scutellaris* has a more restricted geographic territory that covers Papua New Guinea, Tonga, Southeast Asia, the South Pacific [11], Australia [12] and central Thailand [13]. It has long been considered as a potential vector of the dengue virus in Papua New Guinea [14]. It was also incriminated as a dengue virus vector during a huge endemic of dengue virus serotype 2 in 2005 at the Torres Strait in Australia, where *Ae. aegypti* was absent [12], and as a possible vector of the sylvan dengue fever in Bangkok, Thailand [13].

The fourth mosquito species that has been found in Thailand during our entomological surveillance activities, *Verrallina (Ver.) dux*, is attracted by light and feeds on humans, but has never been reported as a vector of any diseases. It is a predominant species in the mangrove forests of Vietnam [15] and the Philippines [16]. In February 2019, *Ve. dux* was collected in the mangrove forest that had been reported previously as the breeding place of *Ae. scutellaris* [6]; both *Ve. dux* and *Ae. scutellaris* reproduce in brackish water.

External morphology at different levels of development has long been the gold standard for taxonomic identification of mosquitoes [11,17,18,19]. The morphological species determination of adults is generally satisfactory, except in two main situations: (i) some adult morphologies are so similar that they are deemed “isomorphic” [20], “sibling” [21] or “cryptic” species [22], and (ii) field mosquitoes may be damaged by the capture device or during transportation to the laboratory, losing the few or the only morphological character allowing their reliable identification [23].

Genetic techniques of mosquito identification represent a valuable tool for these situations [6,19,24], but the recently developed modern morphometric approaches, including landmark-based and outline-based techniques, are increasingly suggested to be efficient complementary diagnostic tools [25,26], and represent non-traumatic, low-cost and frequently accurate discrimination approaches [27]. These methods are applied after a wing preparation procedure involving slide mounting and imaging, skills common among entomologists and which do not pose technical issues [6,23].

Geometric morphometrics of mosquitoes has previously been used to distinguish between genera [28], between species within the same genus [6,23,29,30], between populations of a species [31,32], and between sexes of a species [29,33]. Recently, this method was used by our group to discriminate various organisms as diverse as liver flukes [34], chigger mites [35] and fireflies [23].

The four species of mosquito collected are not sibling species, but they do pose identification problems when partially damaged, especially between *Ae. albopictus* and *Ae. scutellaris*. The latter have no known clear-cut diagnostic traits unless specimens are perfectly preserved. Moreover, larvae and adults of both species are also very similar, and misidentification occurs frequently [2,11,18]. Our sample also contains species which are easier to recognize on morphological grounds, such as *Aedes* ssp. versus *Ve. dux.* We expect that wing metric properties allow clear-cut distinction, especially for *Ve. dux*, a species belonging to a separate genus than *Aedes.*

The three *Aedes (Stegomyia)* species of our sample have recently been examined by both genetic and morphometric techniques [6], including the outline-based approach used here. In this study, we use the shape of the various contours offered by the mosquito wing, not only its external border, but also its various internal cells. Our study was designed to determine whether the taxonomic signal of the wing is spread equally among various internal cells.

## 2. Materials and Methods

### 2.1. Study Area

The four species of mosquito were collected as larvae in various areas of Thailand between 2009 and 2019. Mosquitoes were reared and maintained in the laboratory under the same environmental conditions, and submitted to morphometric analyses at different generational times (Table 1).

*Aedes aegypti* was collected from Bangkhae district (Bangkok province) (13°41′43.6″ N, 100°23′05.1″ E). *Ae. albopictus* was collected from Kanchanaburi Province, 129 km from Bangkok city (14°12′16.2” N, 99°07′58.5″ E). *Ae. scutellaris* was collected from Phasi Charoen (Bangkok province) (13°43′19.8″ N, 100°26′09.2″ E), and *Ve. dux* was collected from the mangrove forest at Bang Pakong (Chachoengsao Province) (13°28′25.0″ N, 100°52′19.9″ E).

### 2.2. Mosquito Colonization

The *Ae. albopictus* and *Ae. scutellaris* were collected between 2009 and 2019 (Table 1) and maintained in the laboratory (Department of Medical Entomology, Faculty of Tropical Medicine, Mahidol University). *Aedes aegypti* and *Ve. dux* was collected more recently from the field, and maintained until the F3 generation before mounting of wings for identification. All four species were identified by external morphology of two-day-old emerging mosquitoes to avoid losing the scale. We used the taxonomic keys of Huang (1972) and Rattanarithikul et al. [2,11].

Rearing conditions of all insects in our laboratory (Department of Medical Entomology, Faculty of Tropical Medicine, Mahidol University) were as follows: 27 °C ± 2 °C and 60% ± 10% relative humidity, and a natural light cycle until adult emergence. Larvae were reared in plastic trays with filtered water, but *Ve. dux* larvae from mangrove forests were reared in filtered water mixed with their natural breeding water. Larvae were provided with 1 mL of fish food solution daily. Pupae were transferred to 30 × 30 × 30 cm^3^ cages to facilitate emergence.

### 2.3. Wings Preparation for Geometric Morphometric Analysis

The right and left wings were dissected and mounted using Hoyer’s medium (mixed from Arabic gum, chloral hydrate, glycerin and distilled water) on glass microscope slides. Each slide was photographed by a Nikon DS-Ri1 SIGHT digital camera connected to a Nikon AZ 100 M stereo-microscope (Nikon Corp., Tokyo, Japan) with the scale apparent on the photograph. The right wing was used, except in case of damage, when the left wing was used instead.

**Figure 1:**
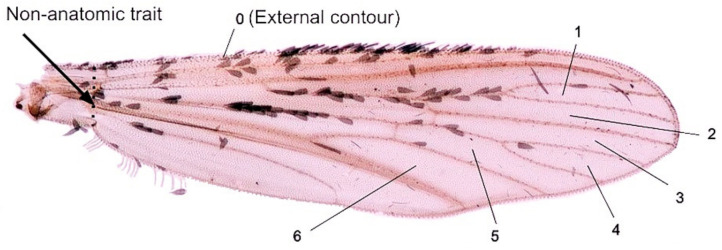
Seven contours digitized on the wing for outline-based geometric morphometric analysis. Cell 0, the external contour of the wing; Cell 1, between veins R2 and R3; Cell 2, delimited by veins R2+3, R3 and R4+5 and rm; Cell 3: delimited by veins R4+5, M1, M1+2 and rm; Cell 4, between veins M1 and M2; Cell 5, delimited by veins M1+2, M2 and M3+4; Cell 6 between M3+4 CuA and mcu. The nomenclature of veins follows Rattaranaritikul et al. 2010 (p. 71). The arrow shows the small part of the external contour which was artificially joined to obtain a completely close outline.

The external contour (cell 0) and the contour of six internal cells (cells 1 to 6) were digitized (Figure 1) using computer-assisted manual digitization (see morphometric software).

### 2.4. Analyses

#### 2.4.1. Size and Shape

**Figure 2:**
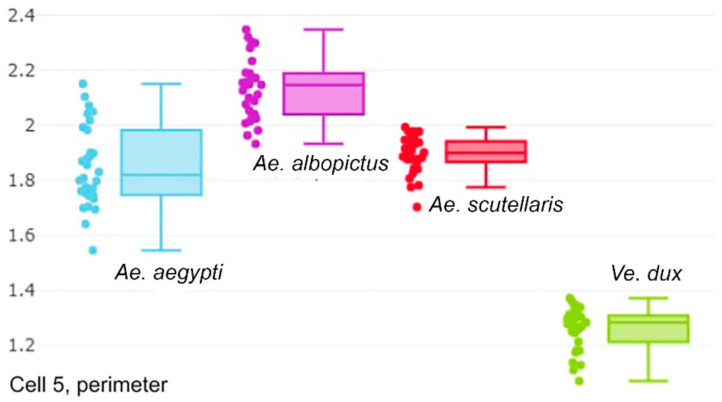
Quantile boxes showing the perimeter of the fifth cell (mm) derived from outline-based geometric morphometrics analysis. Each box shows the group median that separates the 25th and 75th quantiles. From left to right: *Ae. aegypti*; *Ae. albopictus*; *Ae. scutellaris* and *Ve. dux*. The other contours (not shown) showed almost exactly the same interspecific variation: the non-overlapping smaller size of *Ve. dux* versus the other species, and the trend for larger sizes in *Ae. albopictus*.

Elliptic Fourier analysis (EFA) [36] was used to describe the shape of the contour and its size. In this approach, the contour is deconstructed in terms of sine and cosine curves of successive frequencies, called harmonics, with each harmonic containing four coefficients. The removal of the size effect was obtained by dividing the coefficients by the semi-major axis of the first ellipse. However, for presenting a more readable estimate of size, we used the perimeter of each contour, which was highly correlated to the semi-major axis. The size variation amongst the four species was illustrated for cell 5 contour (Figure 2). For both metric properties, i.e., size and shape, statistical comparisons were non-parametric ones based on random permutations (1000 cycles) between groups. The repeatability score [37] was computed as an indirect estimate of measurement error.

#### 2.4.2. Validated Classification

The level of taxonomic information likely to be associated with each contour was measured by the total score of correctly assigned wings after validated classification. The latter was performed using the Mahalanobis distance method, wherein each individual was assigned to the species to which it had the shortest distance. To improve the validity of the method, each individual to be identified was previously removed from the total sample, so that its own metric properties could not influence the classification model; this procedure is known as “validated classification”, as well as “cross-checked classification” or “jackknife classification” [38].

### 2.5. Morphometric Software

We used two packages, the CLIC package version 97 [25], available at (https://xyom-clic.eu, accessed on 16 August 2020), and the recent online morphometric package, XYOM (https://xyom.io, accessed on 16 August 2020) [39]. Computer-assisted manual digitization was performed using XYOM software, which allowed an increase in the number of pseudo-landmarks by automatically adding points between those digitized by the user, provided they fall exactly on the contour. This process was under visual control, and permitted an increase in shape capture.

## 3. Results

Wings belonging to 120 mosquitoes of four species were digitized (30 wings per species): *Ae. aegypti*, *Ae. albopictus*, *Ae. scutellaris* and *Ve. dux.* The repeatability score for size was always above 99%, while it ranged from 80% to 89% for shape.

### 3.1. Wing Size Analysis

Wing size was illustrated by the perimeter (Figure 2). *Aedes albopictus* presented the largest average wing size, while *Ve. dux* presented the smallest (Table 2). This pattern was observed for each contour.

### 3.2. Wing Shape Analysis

The Mahalanobis distances were computed from the external outline, cell 2 and cell 5 were the only ones statistically different between the four species (*p* < 0.05).

#### 3.2.1. Comparing the Taxonomic Information of Different Cells

Cells had consistently different discriminating power, but the external contour (cell 0) never provided the best total score. The least informative contour was cell 6 (see Figure 1), which was obvious in most comparisons (Table 3, Table 4 and Table 5, second column). According to the groups included in the comparisons, the scores of cell 6 ranged from 58% between genera (Table 5, second column) to 92% between *Ae. albopictus* and *Ve. dux (*Table 5). Over the total of 9 comparisons (Table 3, Table 4 and Table 5), the average taxonomic information of cell 6 reached 69%, while the most informative cell (cell 5) reached an average of 91%.

For the external contour and the six internal ones, the following comparisons were performed: (i) A global reclassification of the four species (Table 3), (ii) a global reclassification of the three *Aedes* species (Table 4) and (iii) all possible pairwise reclassifications (Table 5).

The global reclassification of the three *Aedes* species (Table 4) allowed a direct comparison of our work to that performed previously on the same species by Sumruayphol et al. (2016) [6]. For this three-species reclassification, the factor map of the two first discriminatory factors was shown.

Each of the 9 comparisons (Table 3, Table 4 and Table 5) was performed separately for each of the 7 contours (cell 0 to cell 6), totaling 63 validated classifications. All of these (Table 3, Table 4 and Table 5) were performed using the Mahalanobis distance as derived from shape variables, thus tentatively excluding size variation.

**Figure 3:**
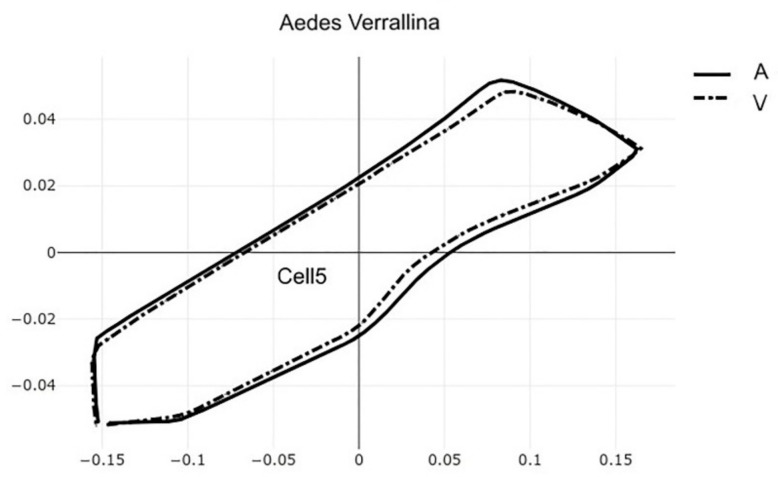
Superposition of size-free contours of cell 5 (see Figure 1) showing shape differences between two genera: *Aedes ssp.* (averaging *Ae. aegypti, Ae. albopictus* and *Ae. scutellaris*) and *Ve. dux* (dashed traits). See also Figure 7, Figure 8 and Figure 9.

**Figure 4:**
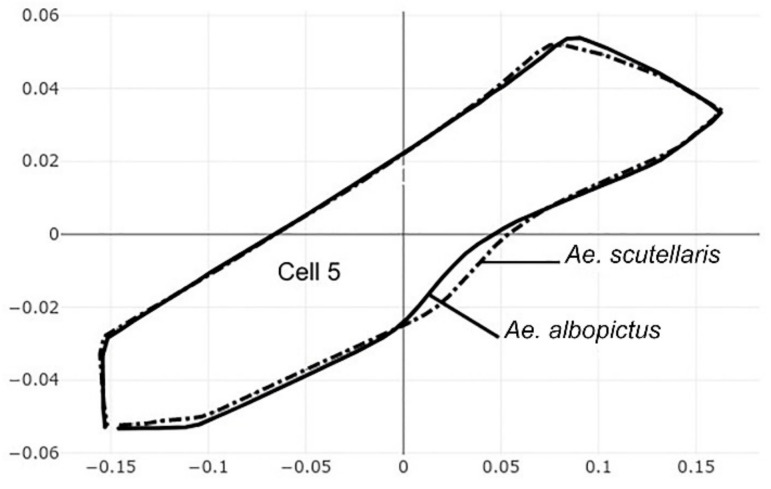
Superposition of size-free contours of cell 5 (see Figure 1) showing shape differences between two species: *Ae. albopictus* (solid line) and *Ae. scutellaris* (dashed traits).

**Figure 5:**
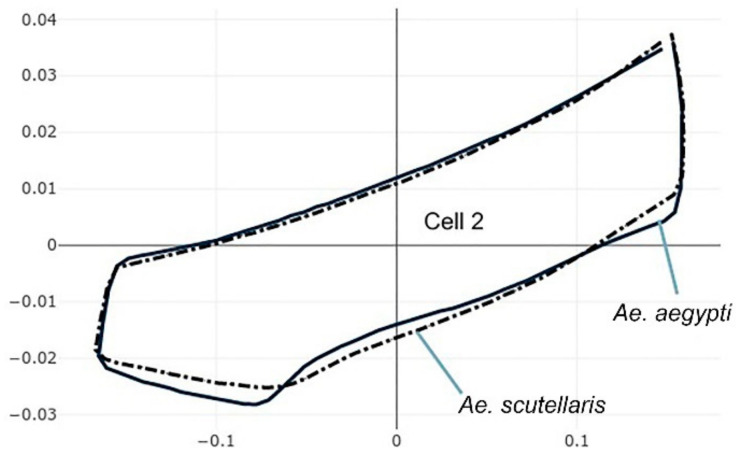
Superposition of size-free contours of cell 2 (see Figure 1) showing shape differences between two species: *Ae. aegypti* (solid line) and *Ae. scutellaris* (dashed traits).

**Figure 6:**
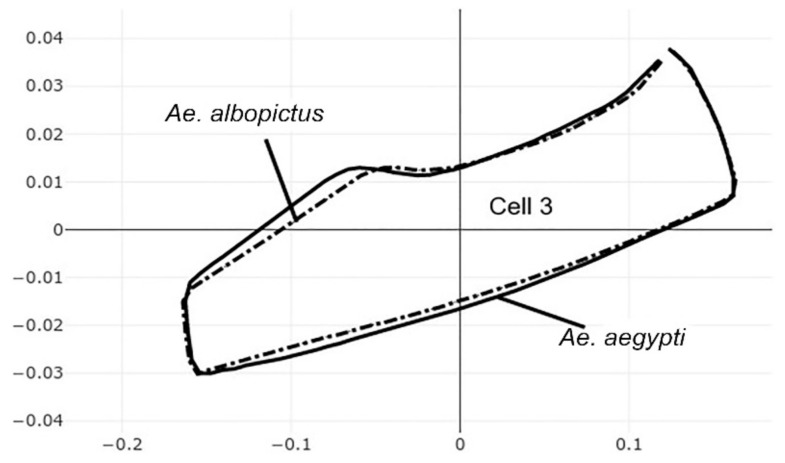
Superposition of size-free contours of cell 3 (see Figure 1) showing shape differences between two species: *Ae. aegypti* (solid line) and *Ae. albopictus* (dashed traits).

**Figure 7:**
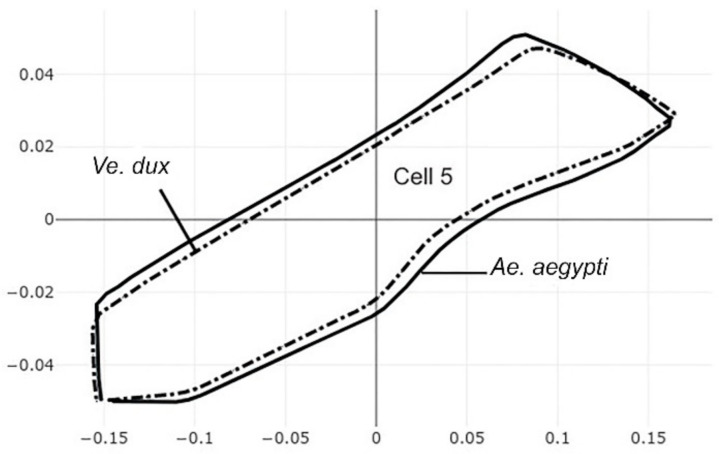
Superposition of size-free contours of cell 5 (see Figure 1) showing shape differences between two species, as well as two genera: *Ae. aegypti* (solid line) and *Ve. dux* (dashed traits).

**Figure 8:**
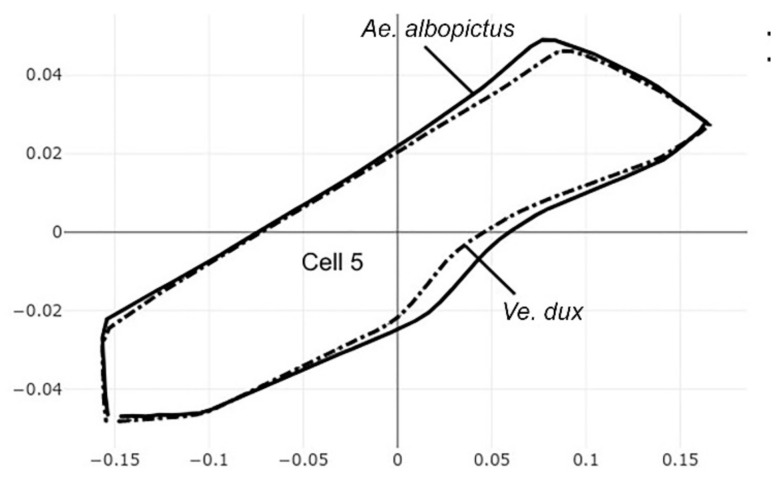
Superposition of size-free contours of cell 5 (see Figure 1) showing shape differences between two species, as well as two genera: *Ae. albopictus* (solid line) and *Ve. dux* (dashed traits).

**Figure 9:**
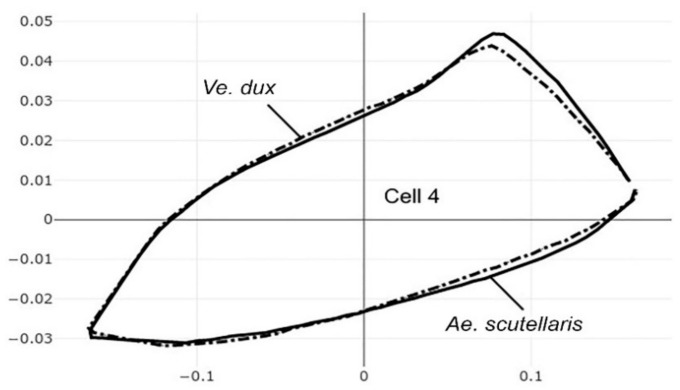
Superposition of size-free contours of cell 4 (see Figure 1) showing shape differences between two species, also two genera: *Ae. scutellaris* (solid line) and *Ve. dux* (dashed traits).

The pairwise comparisons included the one between the two genera *Aedes (Stegomyia)* and *Verralina (Verrallina)*, with sample sizes of 90 and 30, respectively (Table 5, second column). All remaining pairwise comparisons were performed with equal sample sizes (30). For the pairwise comparisons, the superposition of the most-discriminating cells only was shown to visualize the shape changes from one species to another (Figure 3, Figure 4, Figure 5, Figure 6, Figure 7, Figure 8, and Figure 9).

#### 3.2.2. Reclassifying Four Species

The total scores of correct group assignment were low for cell 6 (31%) and cell 1 (61%); scores were acceptable, but not excellent, for the remaining cells (77% to 84%) (Table 3).

#### 3.2.3. Reclassifying Three Species

**Figure 10:**
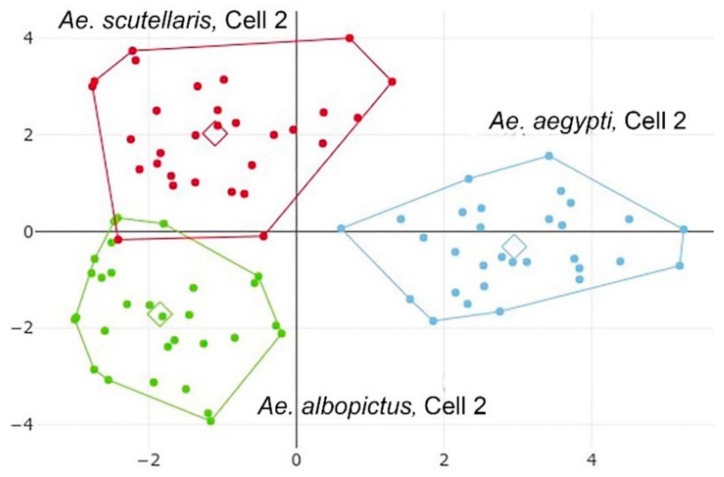
Factor map of the two discriminant factors of shape variables derived from cell 2, the most discriminant cell in the three groups comparisons (see Table 4). *Ae. aegypti* (blue); *Ae. albopictus* (green); *Ae. scutellaris* (red). The first discriminant factor is the horizontal axis.

The same pattern of performance observed for the four-species comparison was confirmed: low scores were observed for cell 6 (36%) and cell 1 (59%), and acceptable (from 72% to 83%) or even very good (88%, from cell 2) scores were observed for the remaining cells (Table 4). The factor map (Figure 10) obtained from cell 2, which was the most informative cell when considering these three taxa, showed a clear tendency of species separation. *Aedes albopictus* and *Ae. scutellaris* were clustered together on one side of the first discriminant factor, and *Ae. aegypti* lay on the other side. This configuration conformed to the previously published phylogenetic tree from Sumruayphol et al., 2016 [6].

#### 3.2.4. Pairwise Reclassifications

Table 5 presents all pairwise reclassifications, including the one between two genera (Table 5, second column). In the intergenera reclassification, the *Aedes* genus is represented by the totality of the three-species sample, with 90 individuals, and the *Verralina* genus contains only one species, the *Ve. dux,* with 30 individuals (see second column of Table 5). Grouping the three species of *Aedes* into one group (n = 90) versus *Ve. dux* (Table 5, second column), the scores did not reach the level of those obtained when comparing the same genera using only one species by genus (Table 5, columns 4, 6 and 7).

Each pairwise comparison had a unique most-discriminating cell: cell 5 to discriminate *Ae. albopictus* from *Ae. scutellaris* (97%, Figure 4), cell 2 between *Ae. aegypti* and *Ae. scutellaris* (98%, Figure 5) and cell 3 between *Ae. aegypti* and *Ae. albopictus* (95%, Figure 6). Excellent scores were reached when the two species compared belonged to different genera (98%, cell 5, Figure 7; 92%, cell 6, Figure 8; 98%, cell 4, Figure 9). 

## 4. Discussion

In this study, we used two *Aedini* genera, *Aedes* (three species) and *Verrallina* (one species). The *Verrallina* species, *Ve. dux*, was examined here by modern morphometrics for the first time. Its morphology is clearly distinct from that of the *Aedes* genus, and as a different genus, it was expected to give us a clearly different, maybe non-overlapping, wing geometry. The other species have been examined previously for the external contour of the wing [6], but not for the internal cells. Internal cells were considered here to determine whether the taxonomic signal of wing contour was spread equally among various internal structures of the wing. We did not use a landmark-based approach for internal cells, as it would be based on too few landmarks (3 to 5, depending on the cell).

Various size and shape differences were disclosed by each wing outline. We showed that some of the shape differences were strong enough to recognize species with high accuracy. We attributed these shape differences to evolutionary divergence, even though there was likely also environmentally induced variation. In our sample, the main sources of possible environmental influence on metric properties could be the following: the number of generations spent in the (same) laboratory, and the water used for larval development [40]. The number of generations before morphometric analyses differed between the four species; therefore, some of the metric differences we found here could be due also to laboratory effects, especially for *Ae. albopictus* and *Ae. scutellaris*, which spent many generations in the laboratory. Previous studies on the influence of the number of generations in the laboratory showed clear changes in the size of the insects, but confirmed the stability of shape [41,42] and of its inheritance [43]. We attempted to maintain similar laboratory conditions for each species: temperature, humidity, food, nutrition, water and container were identical. However, the water solution of *Ve. dux* was different, as a specific salt concentration was maintained for nutrition. To reduce possible laboratory mortality of this species, water from the collected area of origin (mangrove forest) was used in these experiments. Thus, we could not exclude some contribution of the microenvironment to the observed interspecific differences, but these external factors have already been shown to affect size much more than shape [43].

### 4.1. Wing Size Variation

Even within the same species, size may be consistently affected by the number of laboratory generations [41,42], by changes in temperature [44] or humidity [45].

Among the three *Aedes* species, there was considerable overlap of global size, with *Ae. albopictus* tending to be the largest species. Statistical comparisons showed significant differences, excepting the comparison between *Ae. aegypti* and *Ae. scutellaris* (Table 2). In previous studies, *Ae. aegypti* was statistically larger than *Ae. scutellaris* [6]. This apparent discrepancy confirms the lability of size across geographic areas and seasons [23,32].

In our sample, there was a striking difference in size between the two genera, *Aedes* and *Verrallina*. Regardless of the contour considered, *Verrallina* was the smallest species, with no overlapping of size. Such difference is likely to be a generic trait, and could represent per se a simple generic character. However, since size is much more sample-dependent than shape [25], it was excluded from our validated reclassifications.

### 4.2. Wing Shape Variation

Shape as a metric character is much less dependent than size on environmental factors, especially with respect to interspecific differences [25]. Our working hypothesis is that the morphometric variation of shape distinguishing species in our sample was mainly due to evolutionary differences [22,25,46].

#### 4.2.1. Shape Divergence between Species

As expected for a different genus, *Verrallina* (Ver.) *dux* was generally the most discriminated species, recognized at 100% in the four-group comparisons (Table 3). Although some species were adequately recognized when considering the detailed scores in the global comparisons involving three or four groups, the total scores were relatively low: from 31% to 84% in the four-species comparisons (Table 3), and from 36% to 88% in the three-species comparison (Table 4). These total scores as computed from comparisons involving more than two groups were much lower than the ones obtained in pairwise comparisons (80% to 94%, Table 5).

The reclassification scores appeared to depend on two main factors: (i) the number of groups included in the validated reclassification, and (ii) the relative sample sizes of groups involved. For instance, when comparing all four species in one global analysis (Table 3), or three species together (Table 4), the average score of correct attribution for cell 6 was 33.5%, whereas this average was 80% when only two groups were considered (Table 5). The relative sample sizes of the compared groups also influenced the final score: cell 6 was only 58% reliable in recognizing two groups with strongly unequal sample sizes (90 and 30, see second column of Table 5), while the same cell 6 could correctly attribute 84% of species on average when sample sizes were equal (30 and 30) (Table 5, columns 3 to 8). Strongly unequal sample sizes are known to distort Mahalanobis distances [47], which was the distance used in this study for species reclassification.

Considering the external contour of the wing, this study supported the previous results highlighting the outline-based approach to discriminate between the wings of *Ae. aegypti*, *Ae. albopictus* and *Ae. scutellaris* [6]. Our comparison of the three *Aedes* species together (Table 4) yielded scores of total correct recognitions (83%) higher than the ones observed in female mosquitoes by Sumruayphol et al. (2016) (76%) [6]. This could be due to various reasons. As mentioned above, there could be a sampling effect: group sizes were strongly unequal (93, 51 and 45) in the previously published study [6], while they were equal (30, 30 and 30) in our study. Another reason to explain our better performance could be the digitization method. We used an improved manual digitization technique of XYOM (https://xyom.io, accessed on 16 August 2020), a method which increases the number of valid pseudo-landmarks by 10-fold or more; more pseudo-landmarks resulted in a better capture of shape. An additional reason leading to different results could be the different geographic or laboratory origin of the specimens.

#### 4.2.2. Taxonomic Signal among Comparisons

Each cell could be very informative, or not, according to the taxa under comparison. For instance, cell 1 correctly assigned 63% of individuals to their respective species when comparing *Ae. aegypti* and *Ae. albopictus*, whereas the same cell 1 could recognize 95% of individuals when comparing *Ae. scutellaris* and *Ve. dux* (see Table 5).

#### 4.2.3. Taxonomic Signal among Cells

When considering the same comparison, the taxonomic information of different cells could differ widely. The global analysis of all four species highlighted the different taxonomic information associated with each cell, ranging from 31% for cell 6 to 84% for cell 5 (Table 3). This divergence of taxonomic information between cells can be observed also in Table 4 and Table 5. When considering a three-group comparison (Table 4), cells 1 and 6 were obviously uninformative cells (59% and 36%, respectively) relative to the others (ranging from 72% to 88%). The pairwise comparisons (Table 5) show many other examples.

The external contour generally produced slightly lower identification scores than internal cells (see Table 3, Table 4 and Table 5). This weaker taxonomic signal of the largest contour could have a simple technical explanation. Indeed, the contour used here was not a completely anatomic one: the starting point and the ending point, both at the area of junction with the thorax, did not coincide, and were artificially joined by a straight line. This line was obviously not an anatomic part (see arrow on Figure 1). It was, however, not possible to avoid this way of digitizing because each dissected wing was more or less damaged at its articulation with the thorax. Thus, the capture of shape was not complete, even if the loss was very small relative to the remaining part of the external contour. Another explanation could be that the external contour of the wing suffers more biomechanical forces related to flying conditions, constraining its shape to adapt to aerodynamic necessities.

Internal cells are close anatomical contours with no artificial joining of two points, as in the external contour of the wing (Figure 1, see arrow). The unequal taxonomic information of the shape of the various cells examined could not be put in relation with their size. Intuitively, one possible reason for having different recognition power for the same taxa could be related to the complexity of the contour: the more complex the contour, the more substantial the capture of shape. For instance, the most-discriminating cell (cell 5) presented indeed a slightly more complex contour than the others. However, cells as simple as cell 4 produced better scores than cell 5 in some pairwise comparisons (see Table 5 between *Ae. aegypti* and *Ae. scutellaris*, also between *Ve. dux* and *Ae. scutellaris*), and it could even recognize 100% of *Ve. dux* in the four-group comparison (Table 3, detailed score).

Each cell could be very informative, or not, according to the taxa under comparison. Because the taxonomic information of each cell changed unpredictably with the taxa under comparison, there may be some unknown biological explanation. For another group of insects (bees), some variation of the amount of taxonomic information was also observed and remains unexplained [48].

## 5. Conclusions

Our main results can be summarized in two main observations: (i) taxonomic information is not spread equally among cells, and (ii) the taxonomic signal of one or more of internal wing cells can be very high, generally better than the signal associated with the external contour of the wing. The reason for this unequal taxonomic information between internal cells of the same wing was not clear, but it was evidently not related to shape complexity or size of the cells. Moreover, the taxonomic information of a cell can vary greatly according to the taxa under comparison. Of practical interest, even partly damaged wings could contain extractable and accurate taxonomic information, even if it is not actually possible to ascertain which cells should be used.

## Figures and Tables

**Table 1:** Geographic localization and years of capture of the mosquitoes, collected as larvae. From these collections, 30 females were used for morphometric analyses after a different number of generations (F) in the same laboratory.

Species	Locality	Province	Latitude	Longitude	Year	F
*Ae. aegypti*	Bangkhae	Bangkok	13°41′43.6″ N	100°23′05.1″ E	2019	F3
*Ae. albopictus*	Lum Sum	Kanchanaburi	14°12′16.2″ N	99°07′58.5″ E	2009	F49
*Ae. scutellaris*	Phasi charoen	Bangkok	13°43′19.8″ N	100°26′09.2″ E	2011	F33
*Ve. dux*	Bang Pakong	Chachoengsao	13°28′25.0″ N	100°52′19.9″ E	2019	F3

**Table 2:** Perimeter of each cell according to species, and statistical comparisons.

Species	N	Mean (mm)	S.D.	S.E.
**Cell 0**				
*Ae. aegypti*	30	5.60 (4.78–6.45) ^a^	0.45	0.08
*Ae. albopictus*	30	6.10 (5.60–6.54) ^b^	0.27	0.04
*Ae. scutellaris*	30	5.43 (4.87–5.72) ^a^	0.16	0.03
*Ve. dux*	30	3.59 (3.09–3.83) ^c^	0.19	0.03
**Cell 1**				
*Ae. aegypti*	30	1.55 (1.30–1.90) ^a^	0.14	0.02
*Ae. albopictus*	30	1.58 (1.37–1.80) ^a,b^	0.11	0.02
*Ae. scutellaris*	30	1.48 (1.24–1.59) ^a,c^	0.07	0.01
*Ve. dux*	30	0.99 (0.80–1.12) ^d^	0.07	0.01
**Cell 2**				
*Ae. aegypti*	30	2.19 (1.86–2.52) ^a^	0.16	0.02
*Ae. albopictus*	30	2.46 (2.23–2.65) ^b^	0.12	0.02
*Ae. scutellaris*	30	2.20 (1.95–2.30) ^a^	0.07	0.01
*Ve. dux*	30	1.46 (1.24–1.61) ^c^	0.08	0.01
**Cell 3**				
*Ae. aegypti*	30	2.10 (1.81–2.36) ^a^	0.14	0.02
*Ae. albopictus*	30	2.37 (2.12–2.57) ^b^	0.11	0.02
*Ae. scutellaris*	30	2.08 (1.88–2.20) ^a^	0.07	0.01
*Ve. dux*	30	1.41 (1.21–1.55) ^c^	0.08	0.01
**Cell 4**				
*Ae. aegypti*	30	1.22 (1.04–1.46) ^a^	0.10	0.01
*Ae. albopictus*	30	1.27 (1.09–1.45) ^a^	0.09	0.01
*Ae. scutellaris*	30	1.25 (1.06–1.40) ^a^	0.06	0.01
*Ve. dux*	30	0.83 (0.67–0.96) ^b^	0.06	0.01
**Cell 5**				
*Ae. aegypti*	30	1.84 (1.54–2.14) ^a^	0.14	0.02
*Ae. albopictus*	30	2.12 (1.92–2.34) ^b^	0.10	0.01
*Ae. scutellaris*	30	1.89 (1.69–1.98) ^a^	0.06	0.01
*Ve. dux*	30	1.25 (1.06–1.37) ^c^	0.07	0.01
**Cell 6**				
*Ae. aegypti*	30	2.13 (1.75–2.54) ^a^	0.17	0.03
*Ae. albopictus*	30	2.37 (2.13–2.62) ^b^	0.14	0.02
*Ae. scutellaris*	30	2.14 (1.97–2.34) ^a^	0.09	0.01
*Ve. dux*	30	1.42 (1.22–1.58) ^c^	0.09	0.01

Different superscript letters (a, b, c and d) indicate significant differences between species at *p* < 0.05. Mean: average perimeter length calculated using the outline-based method; min: minimum; max: maximum; S.D.: standard deviation and S.E.: standard error.

**Table 3:** Four-species-validated classifications.

	*Ae. aegypti*	*Ae. albopictus*	*Ae. scutellaris*	*Ve. dux*	Total
Cell 0	80% (24/30)	80% (24/30)	93% (28/30)	77% (23/30)	82% (99/120)
Cell 1	43% (16/30)	40% (12/30)	70% (21/30)	80% (24/30)	61% (73/120)
Cell 2	83% (25/30)	77% (23/30)	90% (27/30)	83% (25/30)	83% (100/120)
Cell 3	77% (23/30)	83% (25/30)	70% (21/30)	80% (24/30)	77% (93/120)
Cell 4	70% (21/30)	60% (18/30)	80% (24/30)	100% (30/30)	77% (93/100)
Cell 5	73% (22/30)	83% (25/30)	87% (26/30)	93% (28/30)	84% (101/120)
Cell 6	3% (1/30)	53% (16/30)	7% (2/30)	60% (18/30)	31% (37/120)

For each contour (from cell 0 to cell 6, see Figure 1), detailed scores of validated classifications based on Mahalanobis distances among four species, as derived from shape. The rightmost column contains the total scores of correct species attribution.

**Table 4:** Three-species-validated classifications.

	*Ae. aegypti*	*Ae. albopictus*	*Ae. scutellaris*	Total
Cell 0	80% (24/30)	77% (23/30)	93% (28/30)	83% (75/90)
Cell 1	57% (17/30)	53% (16/30)	67% (20/30)	59% (53/90)
Cell 2	90% (27/30)	83% (25/30)	90% (27/30)	88% (79/90)
Cell 3	77% (23/30)	87% (26/30)	77% (23/30)	80% (72/90)
Cell 4	70% (21/30)	63% (19/30)	83% (25/30)	72% (65/90)
Cell 5	80% (24/30)	83% (25/30)	83% (25/30)	82% (74/90)
Cell 6	43% (13/30)	57% (17/30)	7% (2/30)	36% (32/90)

For each contour (from cell 0 to cell 6, see Figure 1), detailed scores of validated classifications based on Mahalanobis distances, as derived from shape. The rightmost column contains the total scores of correct species attribution.

**Table 5:** Pairwise-validated classifications.

Contours	*Aedes* spp.	aeg	aeg	aeg	alb	alb	scu	Average
	*Ve. dux*	alb	scu	dux	scu	dux	dux	
cell0	90%	77%	77%	85%	93%	83%	90%	*85%*
cell1	82%	67%	82%	75%	77%	87%	95%	*81%*
cell2	84%	92%	98%	85%	87%	85%	85%	*88%*
cell3	86%	95%	83%	83%	92%	90%	97%	*89%*
cell4	94%	63%	95%	92%	78%	90%	98%	*87%*
cell5	96%	92%	88%	98%	97%	92%	93%	*94%*
cell6	58%	83%	80%	87%	77%	92%	82%	*80%*

For each contour (from cell 0 to cell 6, see Figure 1), total scores of validated classifications based on Mahalanobis distances between two groups (detailed scores not shown), as derived from shape. The second column shows the total score of classification *Aedes* spp. (n = 90) and *Ve. dux* (n = 30). The average score of each cell is presented in the last column. The abbreviations of mosquitos’ species are described: *Ae. aegypti* (aeg), *Ae. albopictus* (alb), *Ae. scutellaris* (scu), *Ve. dux* (dux).

## Data Availability

Data is contained within the article.
